# The value of remote continuous nursing based on WeChat short videos for patients with prophylactic ileostomy

**DOI:** 10.3389/fmed.2026.1752820

**Published:** 2026-04-28

**Authors:** Xiaoqiong Chen, Lingnan Li, Guiting Chen, Mingxia Huang, Xinya Wang, Wei Chen, Shuyun Tan

**Affiliations:** 1 Department of General Surgery (Colorectal Surgery), The Sixth Affiliated Hospital, Sun Yat-sen University, Guangzhou, Guangdong, China; 2 Guangdong Provincial Key Laboratory of Colorectal and Pelvic Floor Diseases, The Sixth Affiliated Hospital, Sun Yat-sen University, Guangzhou, Guangdong, China; 3 Biomedical Innovation Center, The Sixth Affiliated Hospital, Sun Yat-sen University, Guangzhou, Guangdong, China

**Keywords:** continuous nursing, ostomy adaptation, prophylactic ileostomy, self-nursing ability, WeChat short videos

## Abstract

**Purpose:**

To explore the effects of remote continuous nursing based on WeChat short videos on the self-nursing ability and adaptation level of patients with prophylactic ileostomy.

**Methods:**

Patients who had undergone radical surgery for colorectal cancer followed with prophylactic ileostomy were enrolled and equally divided into two groups. The control group received routine nursing care of prophylactic ileostomy, while the intervention group received remote continuous nursing through a phased series of WeChat short videos in addition to routine nursing care. Patient mastery level of self-nursing knowledge and adaptability for ostomy were evaluated at discharge, 6 weeks after discharge, and 12 weeks after discharge.

**Results:**

A total of 160 patients with prophylactic ileostomy were included. Compared to patients who only received routine nursing care, routine nursing care plus WeChat video-based remote continuous nursing resulted in a significantly higher level of self-nursing knowledge of ileostomy at discharge (*P* < 0.001). The intervention group also maintained a higher level of ostomy self-nursing knowledge at the 6th week (*P* < 0.001) and 12th week (*P* < 0.001) after discharge. Compared to patients in the control group, the adaptability to an ostomy bag of patients in the intervention group was significantly improved at discharge (*P* < 0.001), the 6th week (*P* < 0.001), and the 12th week (*P* < 0.001) after discharge.

**Conclusion:**

The remote continuous nursing system based on a phased series of WeChat short videos studied can improve the mastery of self-nursing and adaptation to ostomy bags for patients with preventive ileostomy, thereby improving their quality of life.

## Introduction

Colorectal cancer (CRC) represents the third most prevalent malignancy and the second leading cause of cancer-related mortality globally, with its incidence showing a progressive upward trend (1, 2). The current standard treatment for locally advanced colorectal cancer combines radical surgical intervention with neoadjuvant and adjuvant chemoradiotherapy (3). To minimize the clinical severity and infective sequelae of potential anastomotic leakage, approximately 50% of CRC patients undergo prophylactic ileostomy (4, 5). A diverting ileostomy of this nature alleviates endoluminal mechanical pressure and establishes a more favorable environment to facilitate anastomotic healing (6).

Patients with prophylactic ileostomy often suffer from both physiological and psychological pressure due to abnormal odor or fecal leakage (7, 8). Previous studies indicate that about 25% of ileostomy patients experience significant psychological comorbidities including anxiety and depression (9). Compared to colostomy, the higher fluid content and accelerated intestinal motility associated with ileostomy increase susceptibility to complications such as leakage, peristomal dermatitis, granulomatous hyperplasia, and stoma prolapse (10, 11). However, recent studies suggest that discharged patients often demonstrate insufficient knowledge regarding self-care protocols and appropriate nursing methods for ileostomy (12, 13). This knowledge gap may stem from limited preoperative education time and inadequate training during hospitalization, with reported complication rates ranging from 16.3 to 53.8% (14). Thus, most patients with ileostomy require professional post-discharge care guidance, constituting the essential concept of continuous nursing (15).

Current continuous nursing protocols for prophylactic ileostomy predominantly employ telephone consultations and text messaging, though some institutions have developed continuous nursing systems that provide ileostomy nursing consultation and home visits (16). However, these conventional approaches remain constrained by temporal and spatial limitations while requiring substantial human resources. Remote guidance based on communication applications such as WeChat can provide new approaches for continuous nursing. Previous study has constructed a WeChat-based nursing model that can improve ostomy self-management competence and reduce the incidence of relative complications (17). However, these text-based communication systems still present challenges including patients ignoring information and misunderstanding complex procedures.

In this study, we develop a remote continuous nursing system for patients with prophylactic ileostomy based on a prospective clinical trial. Our continuous nursing system generates a series of structured WeChat short videos to facilitate convenient remote continuous nursing. The therapeutic efficacy of this phased, longitudinal nursing intervention was systematically evaluated by comparative analysis of self-nursing ability, stoma adaptation, and quality of life among patients with prophylactic ileostomy.

## Materials and methods

### Study design

This study prospectively enrolled patients with rectal cancers who underwent radical surgery and prophylactic ileostomy in the Sixth Affiliated Hospital of Sun Yat-sen University from June, 2019 to May, 2021. The patients who were eventually included in this study were randomly divided into two groups via a random number method. The Institutional Review Board of the Sixth Affiliated Hospital of Sun Yat-sen University reviewed and approved this study’s protocol. Informed consent was obtained from all participants.

### Participants

All patients included in this study were elective cases who underwent radical surgery for mid-to-low rectal cancer (with or without a history of local radiotherapy). The primary surgical procedures included Laparoscopic or Open Dixon procedure, Parks procedure, and Bacon procedure. The criteria for inclusion in the study were as follows: (a) pathological diagnosis of colorectal cancer and prophylactic ileostomy following radical surgery; (b) aged 18–80 years old; (c) availability for self-care; and (d) access to a smartphone and use of WeChat. The exclusion criteria consisted of: (a) the presence of other types of malignant tumors; (b) serious chronic diseases; and (c) functional disability of the hands or the inability to express subjective desires.

### Construction of the WeChat short video framework

A series of short WeChat videos was designed and filmed based on the consensus for “Minimum Standards for Discharge of Ostomy Patients” reached by the Wound, Ostomy, and Continence Nurses Society (18). Our series of short videos included seven areas of content: (a) knowledge of ostomy bag replacement and related skills; (b) detailed procedures for ostomy bag replacement; (c) assessment of ostomy and peri-ostomy skin conditions; (d) assessment of excrement conditions; (e) dietary precautions; (f) ostomy appliances and access approaches; and (g) approaches for ostomy nursing from professional therapists.

In consideration of patients’ psychological acceptance of ileostomy and willingness to perform self-nursing in different stages after discharge, WeChat short videos were released every week for patients according to the framework shown in Table 1. Briefly, the series was divided into 12 stages, for a total of 12 weeks, with a duration of ≥ 10 min every week. The framework and delivery timing were standardized to align with the typical clinical trajectory including early (weeks 1–3), middle (weeks 4–9) and late stage (weeks 10–12). while the content was standardized, the WeChat platform also enabled individualized management by allowing patients to review specific videos as needed and communicate directly with ostomy therapists for personalized guidance.

**TABLE 1:** The framework of WeChat short videos series.

Time point	Subject	Content	Modality	Video duration
At discharge	Fill in the form	Baseline Characteristic for patients with prophylactic ileostomy	Text	
1–5 weeks after discharge	Replace the ostomy bag (1–3 weeks)	1. Brief introduction of ostomy bag replacement	Video	5–10 min
		2. Detailed demonstration of ostomy bag replacement	Video	5 min
		3. Attention on particulars that are easily overlooked	Video	5–10 min
	Daily Care (4–5 weeks)	4. Effects of diet on stool characteristics	Video	5–10 min
		5. Instruction for daily life	Video	5–10 min
6–9 weeks after discharge	Warm reminder (6th week)	Reminder to watch video via phone or message	Text	
	Ileostomy-related knowledge (6th week)	6. Introduction of auxiliary products for ileostomy and common complications of ileostomy	Video	5–10 min
	Daily care (7th week)	7. Prevention of ileostomy-related complications	Video	5–10 min
	Telephone follow-up		Telephone	5–10 min
	Interaction with patients (8th week)	Fill in the ileostomy Self-Nursing Knowledge Questionnaire	Text	
		8. Interviews for patients with ileostomy about social activities	Video	5 min
	Tips (9th week)	9. Social activities	Video	5 min
10–12 weeks after discharge	Daily Life	10. Dietary considerations	Video	5 min
		11. Healthy Lifestyle	Video	5 min
		12. Things to note when traveling	Video	5 min
	Return to hospital for follow-up	Fill in the Ostomy Patient Adaptation Scale	Text	

### Ileostomy nursing treatments

Patients in the control group received routine ileostomy nursing, including preoperative explanation of the necessity of prophylactic ileostomy and postoperative demonstration of ostomy bag replacement and precautions. The ability of patients to replace their own ostomy bags was assessed, and guidance as to ostomy-care products and related complications was provided at discharge. Compared to the control group, patients in the intervention group received additional remote continuous nursing through the WeChat short videos we developed as detailed in Table 1. Patients were guided to follow the WeChat subscription account of ileostomy nursing during hospitalization. The ostomy therapist then taught patients how to view videos and chat messages in WeChat. The framework of the WeChat short videos was also informed for patients prior to discharge. Standard protocol for stoma closure at our institution occurs approximately 3 months postoperatively. Closure may be delayed due to adjuvant chemotherapy or unhealed anastomotic issues.

### Endpoint evaluation and measurement

To evaluate patients’ abilities with respect to ostomy bag self-nursing, we formulated the “Ileostomy Self-Nursing Knowledge Questionnaire” (Supplementary Data 1) in reference to the “Guidelines for Ileostomy Nursing and Rehabilitation,” “Best Nursing Practice: Discharge Plan for New Stoma Patients,” and “Modern Wound and Stoma Clinical Nursing Practice” (19). Our “Ostomy Patient Adaptation Scale” (Supplementary Data 2) was formulated in reference to Simmons’s research in order to assess patient adaptation to ostomy bags (20).

The detailed criteria of “Ileostomy Self-Nursing Knowledge Questionnaire” and “Ostomy Patient Adaptation Scale” were listed in Table 2. Baseline clinical characteristics of all patients enrolled in this study were acquired prior discharge, and all patients finished the “Ileostomy Self-Nursing Knowledge Questionnaire” and “Ostomy Patient Adaptation Scale” 1 day before discharge. After discharge, patients were followed-up for assessment of ostomy bag self-nursing ability and adaptation to wearing an ostomy bag at the 6th and 12th weeks. The reliability and validity of the questionnaires were evaluated when patients of both groups finished them at discharge.

**TABLE 2:** Detailed criteria for end point evaluation.

Type	Score	Grade
Questionnaire of Ileostomy Self- Nursing Knowledge	<40	Poor mastery of self-nursing knowledge
	40–59	General mastery of self-nursing knowledge
	60–80	Good mastery of self-nursing knowledge
Ostomy Patient Adaptation Scale	<40	Low adaptation level
	40–59	Medium adaptation level
	60–80	High adaptation level

### Statistical analysis

All statistical analysis was conducted with *R* software version 3.6.2,^1^ statistical variables were categorized into continuous and categorical variables. Categorical data were expressed as frequency (n) and percentage (%), and comparisons between groups were made using the *chi-squared* test. Continuous variables following a normal distribution were presented as mean (standard deviation), while those not following a normal distribution were expressed as median (interquartile range). For continuous variables, unpaired *t*-tests were used for normally distributed data, and the *rank-sum* test (*Mann–Whitney U* test) was used for skewed data. For all tests two-sided *P*-values < 0.05 were assumed to indicate statistically significant results.

## Results

### General patient information

This study enrolled 160 patients, and all patients were placed randomly into the control group or the intervention group, with each group having 80 members. There was no significant difference in age between the two groups (58.16 ± 11.69 vs. 55.36 ± 11.86, *P* = 0.14).

### Evaluation of the reliability and validity of the questionnaires

For questionnaire reliability, Cronbach’s α was calculated, and the Component Indicator of Total Credibility (CITC) was used to evaluate the performance of each question. All the questions in the Ileostomy Self-Nursing Knowledge Questionnaire were found to be reliable, with a Cronbach α of 0.734 (Table 3). For the Ostomy Patient Adaptation Scale, after excluding questions 5 and 8, which potentially impaired the reliability, the reliability reached 0.705 and was thus deemed appropriate for subsequent analysis.

**TABLE 3:** The evaluation of reliability for questionnaires.

Type	Question	CITC	Cronbach α
Questionnaire of Ileostomy Self-Nursing Knowledge	Question1	0.821	0.734
	Question2	0.791	
	Question3	0.788	
	Question4	−0.267	
	Question5	0.653	
	Question6	−0.184	
	Question7	0.817	
	Question8	−0.218	
	Question9	0.764	
	Question10	0.119	
	Question11	0.712	
	Question12	0.712	
	Question13	0.759	
	Question14	−0.233	
	Question15	−0.393	
	Question16	−0.276	
	Question17	0.713	
	Question18	−0.279	
	Question19	−0.756	
	Question20	−0.366	
	Question21	0.681	
	Question22	0.638	
	Question23	0.723	
	Question24	−0.411	
	Question25	−0.725	
	Question26	0.78	
	Question27	−0.36	
	Question28	0.745	
	Question29	−0.287	
	Question30	−0.224	
	Question31	−0.272	
Ostomy Patient Adaptation Scale	Question1	0.52	0.705
	Question2	0.322	
	Question3	0.521	
	Question4	0.261	
	Question6	−0.14	
	Question7	−0.253	
	Question9	−0.493	
	Question10	0.668	
	Question11	0.681	
	Question12	0.88	
	Question13	0.274	
	Question14	0.206	
	Question15	−0.116	
	Question16	−0.111	
	Question17	0.782	
	Question18	0.144	
	Question19	−0.006	
	Question20	0.719	

For validity, we first assessed the communality of each question in the questionnaires (Table 4). All the remaining questions had eligible communality in both the Ileostomy Self-Nursing Knowledge Questionnaire and the Ostomy Patient Adaptation Scale. The Kaiser-Meyer-Olkin (KMO) index, which measures sampling adequacy, was 0.899 and 0.858 for the questionnaire of Ileostomy Self-Nursing Knowledge Questionnaire and the Ostomy Patient Adaptation Scale, respectively (Table 5). Furthermore, both questionnaires passed the Bartlett test (*P* < 0.001), confirming their validity.

**TABLE 4:** The evaluation of communality for questionnaires.

Questionnaire	Question	communality
Ileostomy Self-Nursing Knowledge Questionnaire	Question 1	0.83
	Question2	0.748
	Question3	0.792
	Question4	0.676
	Question5	0.688
	Question6	0.699
	Question7	0.81
	Question8	0.789
	Question9	0.767
	Question10	0.624
	Question11	0.699
	Question12	0.692
	Question13	0.747
	Question14	0.648
	Question15	0.614
	Question16	0.764
	Question17	0.739
	Question18	0.665
	Question19	0.714
	Question20	0.503
	Question21	0.669
	Question22	0.572
	Question23	0.613
	Question24	0.68
	Question25	0.66
	Question26	0.738
	Question27	0.694
	Question28	0.717
	Question29	0.512
	Question30	0.733
	Question31	0.525
Ostomy Patient Adaptation Scale	Question1	0.905
	Question2	0.691
	Question3	0.93
	Question4	0.854
	Question6	0.67
	Question7	0.803
	Question9	0.652
	Question10	0.731
	Question11	0.838
	Question12	0.909
	Question13	0.702
	Question14	0.78
	Question15	0.788
	Question16	0.752
	Question17	0.834
	Question18	0.794
	Question19	0.83
	Question20	0.908

**TABLE 5:** The KMO and Bartlett’ test for questionnaires.

Test		Ileostomy Self-Nursing Knowledge Questionnaire	Ostomy Patient Adaptation Scale
KMO		0.899	0.858
Bartlett’ test	Chi-square approximation	3637.001	3035.217
	df	465	153
	*P*	<0.001	<0.001

### Analysis of self-nursing knowledge mastery

**FIGURE 1:**
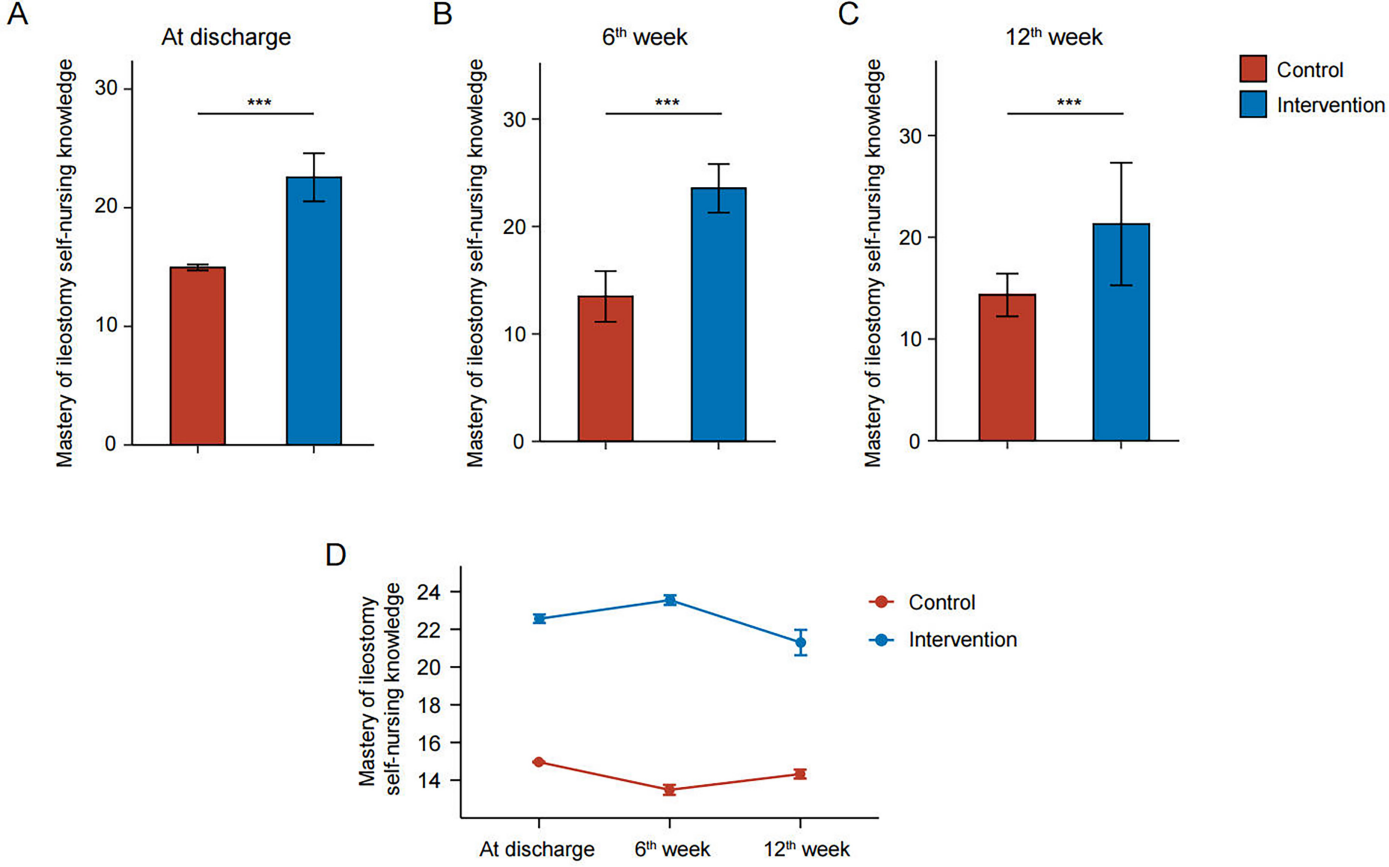
Patients’ levels of self-nursing knowledge for ileostomy in the intervention and control groups at discharge **(A)**, 6 weeks after discharge **(B)**, and 12 weeks after discharge **(C)**. Line chart of the level of self-nursing knowledge for ileostomy patients in the intervention and control groups at different time points **(D)**. ***P < 0.001.

As shown in Table 6, compared to patients who received only routine nursing, patients given routine nursing plus WeChat video-based remote continuous nursing had a significantly higher level of self-nursing knowledge for ileostomy (22.56 ± 2.02 vs. 14.96 ± 0.25, *P* < 0.001) at discharge (Figure 1A). Moreover, at the 6th week (23.55 ± 2.26 vs. 13.49 ± 2.36, *P* < 0.001) and 12th week (21.30 ± 6.03 vs. 14.33 ± 2.10, *P* < 0.001) after discharge, patients who received routine nursing plus WeChat video-based remote continuous nursing still maintained a higher ability of ileostomy self-nursing (Figures 1B,C).

**TABLE 6:** Mastery of ileostomy self-nursing knowledge.

Group	N	Mean ± SD		
		At discharge	6th week	12th week
Control	80	14.96 ± 0.25	13.49 ± 2.36	14.33 ± 2.10
Intervention	80	22.56 ± 2.02	23.55 ± 2.26	21.30 ± 6.03
*P*		<0.001	<0.001	<0.001

For patients in the intervention group, knowledge of ileostomy self-nursing increased significantly during the six weeks after discharge (*P* < 0.05), but then decreased by the 12th week (*P* < 0.05). For patients in the control group, however, knowledge of ileostomy self-nursing decreased significantly during the first 6 weeks after discharge (*P* < 0.05) and remained stable until the 12th week (Figure 1D).

### Analysis of stoma adaptation

**FIGURE 2:**
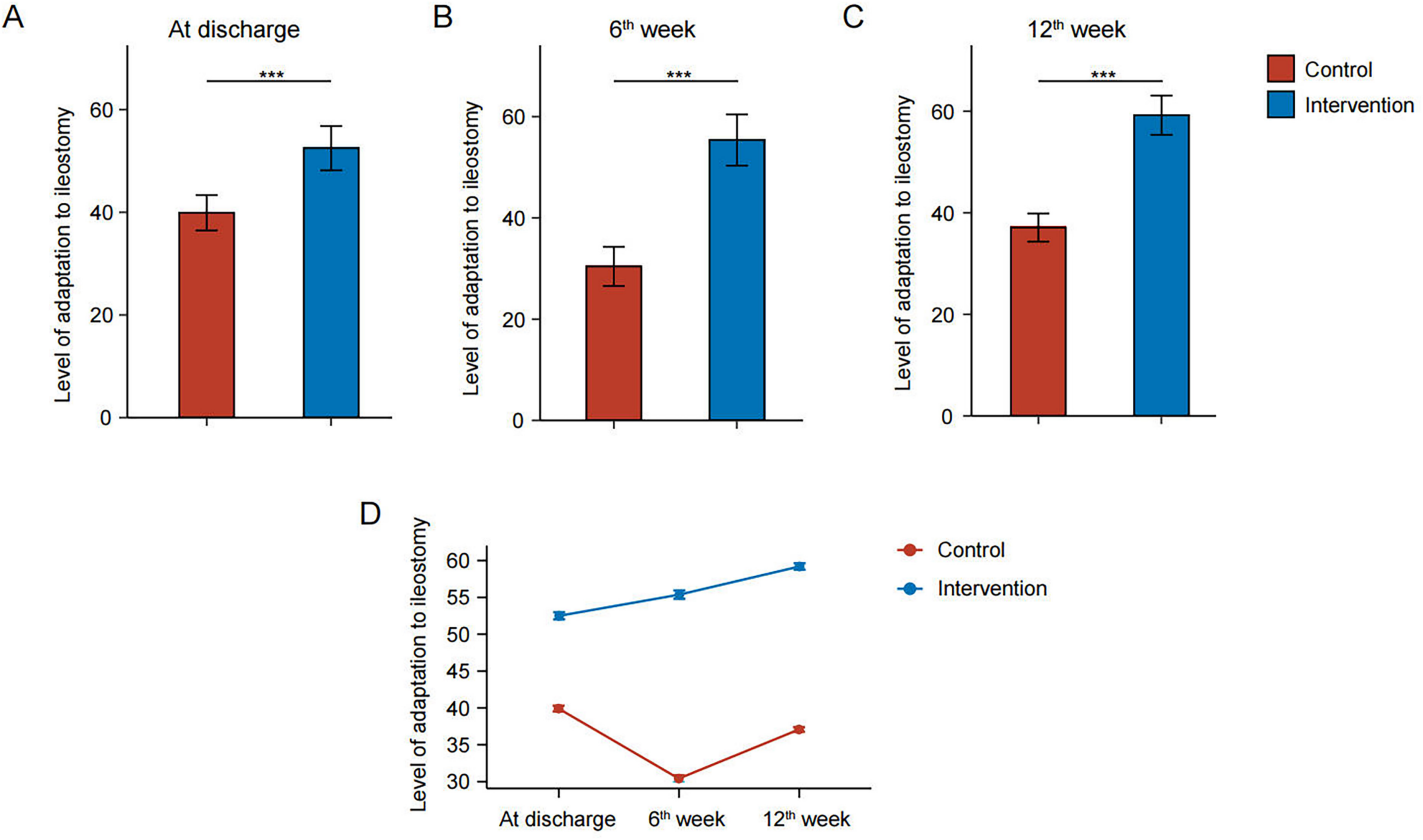
Patients’ levels of adaption to ileostomy bags in the intervention and control groups at discharge **(A)**, 6 weeks after discharge **(B)**, and 12 weeks after discharge **(C)**. Line chart of the level of adaption to ileostomy bags for ileostomy patients in the intervention and control groups at different time points **(D).** ***P < 0.001.

As shown in Table 7, patients who received routine nursing plus WeChat video-based remote continuous nursing better adapted to prophylactic ileostomy compared to patients who only received routine nursing (51.55 ± 5.18 vs. 36.90 ± 2.88, *P* < 0.001) at discharge (Figure 2A). The higher level of adaption to ileostomy was also maintained at 6 weeks (53.88 ± 5.74 vs. 23.43 ± 3.91, *P* < 0.001) and twelve weeks (57.90 ± 5.14 vs. 30.44 ± 3.02, *P* < 0.001) after discharge (Figures 2B,C).

**TABLE 7:** Adaptability of ostomy bag.

Group	*n*	Mean ± SD		
		At discharge	6th week	12th week
Control	80	36.90 ± 2.88	23.43 ± 3.91	30.44 ± 3.02
Intervention	80	51.55 ± 5.18	53.88 ± 5.74	57.90 ± 5.14
*P*		<0.001	<0.001	<0.001

For patients in the intervention group, compared to discharge, the level of adaptation to ileostomy kept increasing to the 6th week (*P* < 0.05) and 12th week (*P* < 0.05) after discharge. Notably, for patients in the control group, the level of adaptation to ileostomy initially decreased at the 6th week (*P* < 0.05) after discharge before increasing to a certain extent at the 12th week (*P* < 0.05) (Figure 2D).

## Discussion

In this study, we developed a remote continuous nursing system by generating a series of WeChat short videos that provided guidance for patients with prophylactic ileostomy, and the effects of our remote continuous nursing system were evaluated in a prospective clinical trial. We found that compared to patients who only received routine nursing, patients who received routine nursing plus WeChat video-based remote continuous nursing had a higher level of knowledge of ileostomy self-nursing and were better adapted to ileostomy after discharge.

The observed superiority of our remote continuous nursing system indicated that continuous nursing can more effectively help patients maintain ostomy self-nursing knowledge they learned during hospitalization also support continuous improvement of their self-nursing abilities compared to standard nursing care (21). In addition, analysis of patients’ adaptation levels showed that continuous nursing using our WeChat videos also promoted patients’ adaptability levels for ileostomy even 12 weeks after discharge. Previous studies have already demonstrated a positive impact of continuous nursing on the postoperative care of patients with ostomy (22). For example, Qi’s study (23) conducted a continuous nursing intervention on elderly patients with abdominal wall ostomy after radical surgery of bladder cancers through questionnaire survey and home communication and found that the intervention could relieve patients’ negative emotions and reduce the risk of related complications. Another study further adopted an informative continuous nursing model using the WeChat platform and public accounts to guide patients toward mastering ostomy-related knowledge and improving their quality of life (24). Going further, based on the previous reported benefit of informative continuous nursing models, we developed a remote continuous nursing system with both staged and long-term plans, thereby helping patients to maintain their capabilities for longer after discharge.

We also analyzed the variations in patients’ ostomy self-nursing knowledge mastery at different time points after discharge. In the control group, patients’ levels of ostomy self-nursing knowledge significantly decreased at the 6th week after discharge due to deficient guidance. However, their knowledge of ostomy self-nursing improved at the 12th week after discharge, which indicated long-term self-nursing may renew patients’ ostomy nursing abilities (25). As for patients in the intervention group, the level of ostomy self-nursing knowledge significantly increased by the 6th week after discharge. Interestingly, however, at the 12th week after discharge, although still higher than those of the control group, the levels of ostomy self-nursing knowledge in patients given the WeChat short video continuous nursing had actually declined. This suggested that there may exist peaks in the learning curve of ostomy self-nursing (26), which provided an impetus for us to modify the framework of WeChat short videos and continue experimenting.

When considering adaptation to ostomy, an indicator that reflects a patient’s actual feelings for prophylactic ileostomy, we found that patients who received the WeChat short video continuous nursing achieved better performance. The adaptation level of ostomy of patients in the intervention group was significantly higher than those in the control group throughout the 12 weeks after discharge. In the control group, patients’ adaptation levels of ostomy dramatically decreased at 6 weeks after discharge, which illustrated that deficient continuous nursing may lead to a rapid decline in patient adaptation to ileostomy and highlights the importance of well-developed continuous nursing systems (27). In line with the mastery of ostomy self-nursing knowledge, the adaptation level of ileostomy for patients in the control group rebounded at the 12th week after discharge, however, and this may have been due to patient adaptability gradually improving as self-nursing knowledge improved during the longer period of the initial self-nursing process (11).

This study demonstrates that integrating WeChat short videos into continuous nursing significantly enhances self-care knowledge and adaptation in patients with prophylactic ileostomy, offering a scalable, cost-effective solution to bridge post-discharge care gaps. The visual, phased delivery of content aligns with recovery stages, addressing complications like leakage and dermatitis while supporting psychological adjustment. Given the high smartphone penetration in regions like China, this model is readily implementable, reducing reliance on resource-intensive in-person follow-ups. Clinicians should prioritize integrating such multimedia tools into standard care protocols to sustain engagement and tailor support, ultimately improving long-term patient outcomes and healthcare efficiency.

This research pioneers the validation of a phased, video-based remote nursing model for ileostomy care, demonstrating its sustained efficacy in enhancing post-discharge self-care knowledge and adaptation. Additionally, we establish a replicable framework for evaluating long-term outcomes in ostomy care, advancing methodological rigor in this field.

There are some limitations to our study. Clinically, we observed that the vast majority of patients successfully resolved routine issues through video guidance. However, we acknowledge that video-based nursing has its boundaries. A small minority of patients with complex or novel complications required professional in-person intervention. Our study primarily focused on the holistic gains in self-care mastery and psychosocial adaptation rather than the absolute frequency of complication-related readmissions. Furthermore, a lack of quantitative data and relatively small sample size were needed to be improve in an independent validation with a larger cohort in future work.

## Conclusion

The remote continuous nursing system based on a phased series of WeChat short videos we implemented in this study can improve mastery of self-nursing and adaptation to ostomy bag for patients with preventive ileostomy, thereby improving their quality of life.

## Data Availability

The raw data supporting the conclusions of this article will be made available by the authors, without undue reservation.

## Footnotes

1
http://www.R-project.org

## References

[1] Siegel RL Miller KD Wagle NS Jemal A. Cancer statistics, 2023. *CA Cancer J Clin.* (2023) 73:17–48. 10.3322/caac.21763 36633525

[2] Xie Y Shi L He X Luo Y. Gastrointestinal cancers in China, the USA, and Europe. *Gastroenterol Rep.* (2021) 9:91–104. 10.1093/gastro/goab010 34026216 PMC8128023

[3] Provenzale D Ness RM Llor X Weiss JM Abbadessa B Cooper G et al. NCCN guidelines insights: colorectal cancer screening, version 2.2020. *J Natl Comp Cancer Net.* (2020) 18:1312–20. 10.6004/jnccn.2020.0048 PMC8311627 33022639

[4] Sanne JV Jara EJ Edgar JBF Wendy K Christiaan H Daniel AH et al. The effect of a temporary stoma on long-term functional outcomes following surgery for rectal cancer. *Dis Colon Rectum.* (2023) 67:291–301. 10.1097/dcr.0000000000003009 38127585 PMC10769172

[5] Lightner AL Pemberton JH. The role of temporary fecal diversion. *Clin Colon Rect Surg.* (2017) 30:178–83. 10.1055/s-0037-1598158 PMC5498160 28684935

[6] Peter I Petra G Matúš P Petr V Anton P Pavel Z. Diverting ileostomy in laparoscopic rectal cancer surgery: high price of protection. *Surg Endosc.* (2016) 30:4809–16. 10.1007/s00464-016-4811-3 26902615

[7] Rowe KM Schiller LR. Ileostomy diarrhea: pathophysiology and management. *Proceedings (Baylor University Medical Center).* (2020) 33:218–26. 10.1080/08998280.2020.1712926 32313465 PMC7155987

[8] Babakhanlou R Larkin K Hita AG Stroh J Yeung SC. Stoma-related complications and emergencies. *Int J Emerg Med.* (2022) 15:17. 10.1186/s12245-022-00421-9 35534817 PMC9082897

[9] Moraes JT Borges EL Santos CF da Silva ME de Sá FDS. Prevalence of anxiety and depression in persons with ostomies: a cross-sectional study. *J Wound Ostomy Continence Nurs.* (2020) 47:595–600. 10.1097/won.0000000000000718 33201146

[10] Steinhagen E Colwell J Cannon LM. Intestinal stomas-postoperative stoma care and peristomal skin complications. *Clin Colon Rect Surg.* (2017) 30:184–92. 10.1055/s-0037-1598159 28684936 PMC5498169

[11] Momeni Pour R Darvishpour A Mansour-Ghanaei R Kazemnezhad Leyli E. The effects of education based on the nursing process on ostomy self-care knowledge and performance of elderly patients with surgical stoma. *Nurs Res Pract.* (2023) 2023:2800796. 10.1155/2023/2800796 36644020 PMC9833921

[12] Ngo TD Hawks M Nguyen TTT Nguyen TNH Nguyen HT Mai NTT. Self-care knowledge in patients with intestinal stomas in a selected hospital in the south of Viet Nam: a descriptive cross-sectional study. *Belit Nurs J.* (2023) 9:331–8. 10.33546/bnj.2711 PMC10461151 37645583

[13] Naseh L Shahriari M Hayrabedian A Moeini M. Nurses’ viewpoints on factors affecting ostomy care: a qualitative content analysis. *Nurs Open.* (2023) 10:5261–70. 10.1002/nop2.1764 37084269 PMC10333817

[14] Murken DR Bleier JIS. Ostomy-related complications. *Clin Colon Rect Surg.* (2019) 32:176–82. 10.1055/s-0038-1676995 31061647 PMC6494607

[15] Wei S Li N Li X Qi M. Effect of continuous nursing on wound infection and quality of life in patients with cancer-related stoma: a meta-analysis. *Int Wound J.* (2023) 20:3974–80. 10.1111/iwj.14285 37376826 PMC10681417

[16] Jin Y Tian X Li Y Jiménez-Herrera M Wang H. Effects of continuous care on health outcomes in patients with stoma: a systematic review and meta-analysis. *Asia-Pac J Oncol Nurs.* (2022) 9:21–31. 10.1016/j.apjon.2021.12.006 35528792 PMC9072188

[17] Hao J Xu Y Li H. The value of applying a continuous nursing model based on virtual platforms for patients with colostomy or ileostomy. *Adv Skin Wound Care.* (2023) 36:206–12. 10.1097/01.asw.0000919960.94295.53 36940377 PMC10026954

[18] Colwell JC Kupsick PT McNichol LL. Outcome criteria for discharging the patient with a new ostomy from home health care: a WOCN society consensus conference. *J Wound Ostomy Continence Nurs.* (2016) 43:269–73. 10.1097/won.0000000000000230 27163682

[19] Prinz A Colwell JC Cross HH Mantel J Perkins J Walker CA. Discharge planning for a patient with a new ostomy: best practice for clinicians. *J Wound Ostomy Continence Nurs.* (2015) 42:79–82. 10.1097/won.0000000000000094 25333690

[20] Simmons KL Smith JA Bobb KA Liles LL. Adjustment to colostomy: stoma acceptance, stoma care self-efficacy and interpersonal relationships. *J Adv Nurs.* (2007) 60:627–35. 10.1111/j.1365-2648.2007.04446.x 18039249

[21] McNichol L Markiewicz A Goldstine J Nichols TRA. Cross-sectional survey reporting on the value of patient-centered ostomy programs: a smooth transition after ostomy surgery. *J Wound Ostomy Continence Nurs.* (2022) 49:449–54. 10.1097/won.0000000000000907 36108228 PMC9481286

[22] Liu XJ Han J Su X. Influence of continuous nursing on surgical site wound infection and postoperative complication for colorectal cancer patients with stoma: a meta-analysis. *Int Wound J.* (2024) 21:e14480. 10.1111/iwj.14480 38083831 PMC10958097

[23] Zhang T Qi X. Enhanced nursing care for improving the self-efficacy & health-related quality of life in patients with a urostomy. *J Multidisc Healthcare.* (2023) 16:297–308. 10.2147/jmdh.s394515 36741293 PMC9893841

[24] Zhou L Zhang F Li H Wang L. Post-discharge health education for patients with enterostomy: a nationwide interventional study. *J Global Health.* (2023) 13:04172. 10.7189/jogh.13.04172 38085224 PMC10716631

[25] Liu Y Wang L Zhu L. The impact of stoma management education on the self-care abilities of individuals with an intestinal stoma. *Br J Nurs (Mark Allen Publishing).* (2023) 32:S28–33. 10.12968/bjon.2023.32.6.s28 36952366

[26] Song QF Yin G Guo X Lv X Yu K Liu C. Effects of a self-management program for patients with colorectal cancer and a colostomy: a nonrandomized clinical trial. *J Wound Ostomy Continence Nurs.* (2021) 48:311–7. 10.1097/won.0000000000000779 34186549

[27] Ko HF Wu MF Lu JZ. A randomized control study: the effectiveness of multimedia education on self-care and quality of life in patients with enterostomy. *Int Wound J.* (2023) 20:4244–52. 10.1111/iwj.14326 37488713 PMC10681488

