# Supplementary material for: The value of remote continuous nursing based on WeChat short videos for patients with prophylactic ileostomy
Source: Front Med (Lausanne). 2026 Apr 28;13:1752820. doi: 10.3389/fmed.2026.1752820 (PMC13160747; doi:10.3389/fmed.2026.1752820)
Supplement: Supplementary file 1 [file Table_1.docx]

**Ileostomy Self-Nursing Knowledge Questionnaire**

1. Basic Information: [Matrix Text Question]

[Name]

[Age]

[Gender]

2. Which of the following is an appropriate way to store spare ostomy bag at home? [Single-choice Question]

○ Store in a place directly exposed to sunlight to sterilize them, making them more hygienic for use.

○ Store in a refrigerator or other low-temperature facilities.

○ Store at room temperature in a dry and non-humid environment, without placing heavy objects on top.

3. When is the most appropriate time to replace the ostomy bag? [Single-choice Question]

○ In the morning.

○ After meals.

○ Before sleep.

4. Which of the following sequences for changing the ostomy bag is reasonable? [Single-choice Question]

○ Prepare all the necessary items, cut the baseplate according to previous experience; remove the ostomy pouch; clean the stoma and the skin around the stoma; appropriately apply stoma powder, skin protectant and leak-proof paste; attach a new ostomy bag.

○ Remove the ostomy pouch; clean the stoma and the skin around the stoma; appropriately apply stoma powder, skin protectant and leak-proof paste; measure the size of the stoma and cut the baseplate; attach a new ostomy bag.

○ Prepare all the necessary items; remove the ostomy bag; observe the stoma and the surrounding skin; clean the stoma and the skin around the stoma; appropriately use stoma powder, skin protectant and leak-proof paste; measure the size of the stoma and cut the baseplate; attach a new ostomy bag.

5. How to remove the old ostomy bag? [Single-choice Question]

○ Use one hand to directly tear off the ostomy bag from one side of the stoma baseplate.

○ Press the skin with one hand and gently lift the ostomy bag with the other hand, removing the baseplate from bottom to top.

○ Press the skin with one hand and gently lift the ostomy bag with the other hand, removing the baseplate from top to bottom.

6. After the ostomy bag is removed, if feces keep flowing out during the process of changing the ostomy bag, which handling method is more reasonable? [Single-choice Question]

○ Prepare a large amount of tissue paper. If feces flow out during replacement, wipe them off quickly to keep the skin around the stoma dry.

○ Do not take any action and quickly complete the replacement

○ Roll a piece of gauze into a plug and press it on the stoma, or place a clean small towel on the stoma to prevent feces from contaminating the skin around the stoma.

7. Which of the following stoma manifestations is abnormal? [Single-choice Question]

○ The color is pink or beefy red.

○ Slightly protrudes above the skin surface, shiny and moist.

○ The color is purplish red, ecchymotic red, or black.

8. When changing the ostomy bag, if the skin around the stoma is slightly red for the first time, how should it be handled? [Single-choice Question]

○ Continue with the subsequent steps and ignore it.

○ Apply stoma skin care powder, skin protectant, and/or stoma leak-proof paste to the abnormal skin area.

○ Disinfect with iodine tincture.

9. Which of the following foods can help reduce odors? [Single-choice Question]

○ Asparagus, fish.

○ Cranberry juice, cheese.

○ Spices.

10. Which of the following statements is incorrect? [Single-choice Question]

○ The size of the stoma may change within 6 - 8 weeks after surgery. After this period, when changing the ostomy bag, you can cut the baseplate according to previous experience first and then remove the ostomy pouch.

○ The drainage direction of the ostomy pouch should be determined according to your usual posture.

○ During the entire process, if you are afraid of not doing it well, you can never participate and completely rely on your family members for help every time.

11. In the face of negative emotions, which of the following is not advisable? [Single-choice Question]

○ Worried about the unfriendliness of people around you, isolate yourself and refuse social activities.

○ Actively make friends with people who have had the same experience and exchange nursing experiences and feelings.

○ Talk to family members or medical staff and actively seek solutions.

12. Which of the following choices for daily diet is correct? [Single-choice Question]

○ Drink milk and other dairy products every day to supplement nutrition.

○ Drink enough water every day and mainly have a light, easy-to-digest and balanced diet.

○ According to your own taste, eat fried and spicy food and drink carbonated beverages such as beer.

13. After the wound has healed, you can take a bath. Which of the following statements is correct? [Single-choice Question]

○ If wearing an ostomy bag, you can use waterproof tape to stick the edge of the stoma baseplate.

○ Directly flush the stoma and the edge of the stoma with water.

○ After taking a bath, don't care whether the area around the ostomy pouch is dried or not.

14. Regarding the choice of daily exercise, which of the following is more appropriate? [Single-choice Question]

○ Stay at home and be less active.

○ Spontaneously play basketball or do boxing and other sports with intense collisions with friends.

○ Combine your physical condition and do appropriate exercises, such as walking, square dancing, practicing qigong, etc.

15. Which of the following statements is incorrect? [Single-choice Question]

○ It is best to return to the hospital for a review within 1 month after surgery, and then have regular reviews.

○ When encountering adverse changes in the nature of feces, complications of the stoma and the surrounding skin, and urine leakage that cannot be resolved, you should actively seek medical help.

○ If you feel that the symptoms are mild, don't take it seriously and deal with it on your own.

16. The stoma is a wound and a kind of disease. [Single-choice Question]

○ True

○ False

17. The stoma baseplate is generally changed once every 3-5 days. If there is no leakage, it can be left unchanged until leakage occurs. [Single-choice Question]

○ True

○ False

18. Non-disposable ostomy pouches can be removed at any time, washed, dried, and used until they are damaged. [Single-choice Question]

○ True

○ False

19. It is best to place a nursing pad before changing the ostomy bag to prevent feces from soiling the bed sheets if they are discharged at any time. [Single-choice Question]

○ True

○ False

20. Before removing the old ostomy bag, gently press around the stoma with your hand to prevent feces from overflowing during the replacement. If it is difficult to remove, you can moisten the baseplate with a wet gauze and then remove the ostomy bag. [Single-choice Question]

○ True

○ False

21. After removing the old stoma baseplate, check the stoma and the surrounding skin, the condition of feces, and observe the leaking and dissolved parts of the ostomy bag baseplate. [Single-choice Question]

○ True

○ False

22. The stoma can be gently scrubbed from the inside out with a cotton swab soaked in warm water. [Single-choice Question]

○ True

○ False

23. You can wipe the stoma and the surrounding skin with a disinfectant to prevent infection. [Single-choice Question]

○ True

○ False

24. When there are allergic symptoms such as redness, ulceration, peeling, and itching of the skin around the stoma, apply a thick layer of stoma powder to protect the skin. [Single-choice Question]

○ True

○ False

25. Cut the stoma baseplate according to the size of the stoma, which can be about 0.2 cm larger than the stoma. After proper cutting, you can use your finger to smooth the stoma ring of the baseplate to avoid the uneven edge damaging the stoma. [Single-choice Question]

○ True

○ False

26. Apply a thin layer of leak-proof paste to the edge of the opening on the back of the cut stoma baseplate, align it with the stoma, and stick it on to reduce fecal leakage. [Single-choice Question]

○ True

○ False

27. To avoid excessive stoma discharge, in daily life, you can drink less water to prevent leakage or accidentally getting the bed sheets or clothes wet during replacement. [Single-choice Question]

○ True

○ False

28. Usually, pay attention to observing and recording the amount of fecal discharge. If the flow rate is too high, report it to the medical staff timely. [Single-choice Question]

○ True

○ False

29. If you have a fever, you can take some medicine to control your body temperature and don't need to take it seriously. [Single-choice Question]

○ True

○ False

30. Choose soap or body wash that does not leave residues easily when taking a bath to avoid the residues affecting the stability of the stoma baseplate adhesion. [Single-choice Question]

○ True

○ False

31. When traveling, prepare enough ostomy bags and put them in your carry-on luggage. Also, bring a bottle of mineral water with you in case you need to rinse when emergency. [Single-choice Question]

○ True

○ False

32. Do you need ostomy extended care, such as WeChat follow-up, telephone follow-up, remote video follow-up, etc.? [Single-choice Question]

○ Yes

○ No

***Answer of the Ileostomy Self-Nursing Knowledge Questionnaire***

2. Which of the following is an appropriate way to store spare ostomy bag at home? [Single-choice Question]

○ Store at room temperature in a dry and non-humid environment, without placing heavy objects on top.

3. When is the most appropriate time to replace the ostomy bag? [Single-choice Question]

○ In the morning.

4. Which of the following sequences for changing the ostomy bag is reasonable? [Single-choice Question]

○ Prepare all the necessary items; remove the ostomy bag; observe the stoma and the surrounding skin; clean the stoma and the skin around the stoma; appropriately use stoma powder, skin protectant and leak-proof paste; measure the size of the stoma and cut the baseplate; attach a new ostomy bag.

5. How to remove the old ostomy bag? [Single-choice Question]

○ Press the skin with one hand and gently lift the ostomy bag with the other hand, removing the baseplate from top to bottom.

6. After the ostomy bag is removed, if feces keep flowing out during the process of changing the ostomy bag, which handling method is more reasonable? [Single-choice Question]

○ Roll a piece of gauze into a plug and press it on the stoma, or place a clean small towel on the stoma to prevent feces from contaminating the skin around the stoma.

7. Which of the following stoma manifestations is abnormal? [Single-choice Question]

○ The color is purplish red, ecchymotic red, or black.

8. When changing the ostomy bag, if the skin around the stoma is slightly red for the first time, how should it be handled? [Single-choice Question]

○ Apply stoma skin care powder, skin protectant, and/or stoma leak-proof paste to the abnormal skin area.

9. Which of the following foods can help reduce odors? [Single-choice Question]

○ Asparagus, fish.

10. Which of the following statements is incorrect? [Single-choice Question]

○ During the entire process, if you are afraid of not doing it well, you can never participate and completely rely on your family members for help every time.

11. In the face of negative emotions, which of the following is not advisable? [Single-choice Question]

○ Worried about the unfriendliness of people around you, isolate yourself and refuse social activities.

12. Which of the following choices for daily diet is correct? [Single-choice Question]

○ Drink enough water every day and mainly have a light, easy-to-digest and balanced diet.

13. After the wound has healed, you can take a bath. Which of the following statements is correct? [Single-choice Question]

○ If wearing an ostomy bag, you can use waterproof tape to stick the edge of the stoma baseplate.

14. Regarding the choice of daily exercise, which of the following is more appropriate? [Single-choice Question]

○ Combine your physical condition and do appropriate exercises, such as walking, square dancing, practicing qigong, etc.

15. Which of the following statements is incorrect? [Single-choice Question]

○ If you feel that the symptoms are mild, don't take it seriously and deal with it on your own.

16. The stoma is a wound and a kind of disease. [Single-choice Question]

○ False

17. The stoma baseplate is generally changed once every 3-5 days. If there is no leakage, it can be left unchanged until leakage occurs. [Single-choice Question]

○ False

18. Non-disposable ostomy pouches can be removed at any time, washed, dried, and used until they are damaged. [Single-choice Question]

○ False

19. It is best to place a nursing pad before changing the ostomy bag to prevent feces from soiling the bed sheets if they are discharged at any time. [Single-choice Question]

○ True

20. Before removing the old ostomy bag, gently press around the stoma with your hand to prevent feces from overflowing during the replacement. If it is difficult to remove, you can moisten the baseplate with a wet gauze and then remove the ostomy bag. [Single-choice Question]

○ False

21. After removing the old stoma baseplate, check the stoma and the surrounding skin, the condition of feces, and observe the leaking and dissolved parts of the ostomy bag baseplate. [Single-choice Question]

○ True

22. The stoma can be gently scrubbed from the inside out with a cotton swab soaked in warm water. [Single-choice Question]

○ True

23. You can wipe the stoma and the surrounding skin with a disinfectant to prevent infection. [Single-choice Question]

○ False

24. When there are allergic symptoms such as redness, ulceration, peeling, and itching of the skin around the stoma, apply a thick layer of stoma powder to protect the skin. [Single-choice Question]

○ False

25. Cut the stoma baseplate according to the size of the stoma, which can be about 0.2 cm larger than the stoma. After proper cutting, you can use your finger to smooth the stoma ring of the baseplate to avoid the uneven edge damaging the stoma. [Single-choice Question]

○ True

26. Apply a thin layer of leak-proof paste to the edge of the opening on the back of the cut stoma baseplate, align it with the stoma, and stick it on to reduce fecal leakage. [Single-choice Question]

○ True

27. To avoid excessive stoma discharge, in daily life, you can drink less water to prevent leakage or accidentally getting the bed sheets or clothes wet during replacement. [Single-choice Question]

○ False

28. Usually, pay attention to observing and recording the amount of fecal discharge. If the flow rate is too high, report it to the medical staff timely. [Single-choice Question]

○ True

29. If you have a fever, you can take some medicine to control your body temperature and don't need to take it seriously. [Single-choice Question]

○ False

30. Choose soap or body wash that does not leave residues easily when taking a bath to avoid the residues affecting the stability of the stoma baseplate adhesion. [Single-choice Question]

○ True

31. When traveling, prepare enough ostomy bags and put them in your carry-on luggage. Also, bring a bottle of mineral water with you in case you need to rinse when emergency. [Single-choice Question]

○ True

32. Do you need ostomy extended care, such as WeChat follow-up, telephone follow-up, remote video follow-up, etc.? [Single-choice Question]

○ Yes
