# Supplementary material for: The value of remote continuous nursing based on WeChat short videos for patients with prophylactic ileostomy
Source: Front Med (Lausanne). 2026 Apr 28;13:1752820. doi: 10.3389/fmed.2026.1752820 (PMC13160747; doi:10.3389/fmed.2026.1752820)
Supplement: Supplementary file 2 [file Table_2.docx]

**Ostomy Patient Adaptation Scale**

1. Basic Information:

[Name]

[Hospitalization Number]

[Age]

2. Because of the ostomy, I feel that I will always be a patient. [Single-choice Question]

○ Strongly Agree

○ Agree

○ Uncertain

○ Disagree

○ Strongly Disagree

3. Ostomy care is a very difficult thing. [Single-choice Question]

○ Strongly Agree

○ Agree

○ Uncertain

○ Disagree

○ Strongly Disagree

4. The ostomy limits my range of activities. [Single-choice Question]

○ Strongly Agree

○ Agree

○ Uncertain

○ Disagree

○ Strongly Disagree

5. I often worry that the ostomy will leak, have an odor, or make noise. [Single-choice Question]

○ Strongly Agree

○ Agree

○ Uncertain

○ Disagree

○ Strongly Disagree

6. I still can't accept my ostomy. [Single-choice Question]

○ Strongly Agree

○ Agree

○ Uncertain

○ Disagree

○ Strongly Disagree

7. Because of the ostomy, I often feel anxious. [Single-choice Question]

○ Strongly Agree

○ Agree

○ Uncertain

○ Disagree

○ Strongly Disagree

8. I think the ostomy makes me lose my sexual charm. [Single-choice Question]

○ Strongly Agree

○ Agree

○ Uncertain

○ Disagree

○ Strongly Disagree

9. Since I had the ostomy, I refused to participate in social activities. [Single-choice Question]

○ Strongly Agree

○ Agree

○ Uncertain

○ Disagree

○ Strongly Disagree

10. Because of the ostomy, I feel that my life seems to be out of control. [Single-choice Question]

○ Strongly Agree

○ Agree

○ Uncertain

○ Disagree

○ Strongly Disagree

11. I believe that in the future, I will definitely be able to manage my ostomy well. [Single-choice Question]

○ Strongly Agree

○ Agree

○ Uncertain

○ Disagree

○ Strongly Disagree

12. Despite having a ostomy, my life is still very meaningful. [Single-choice Question]

○ Strongly Agree

○ Agree

○ Uncertain

○ Disagree

○ Strongly Disagree

13. Despite having had ostomy surgery, I still feel that life is very meaningful. [Single-choice Question]

○ Strongly Agree

○ Agree

○ Uncertain

○ Disagree

○ Strongly Disagree

14. I have regarded the ostomy as a part of my body. [Single-choice Question]

○ Strongly Agree

○ Agree

○ Uncertain

○ Disagree

○ Strongly Disagree

15. Despite having a ostomy, I can still participate in various activities. [Single-choice Question]

○ Strongly Agree

○ Agree

○ Uncertain

○ Disagree

○ Strongly Disagree

16. I am very grateful because the ostomy has given me a new life. [Single-choice Question]

○ Strongly Agree

○ Agree

○ Uncertain

○ Disagree

○ Strongly Disagree

17. After having the ostomy, I feel that my life is no longer threatened by the original disease. [Single-choice Question]

○ Strongly Agree

○ Agree

○ Uncertain

○ Disagree

○ Strongly Disagree

18. I can sleep well without worrying about my ostomy. [Single-choice Question]

○ Strongly Agree

○ Agree

○ Uncertain

○ Disagree

○ Strongly Disagree

19. I feel that I have recovered from the ostomy surgery. [Single-choice Question]

○ Strongly Agree

○ Agree

○ Uncertain

○ Disagree

○ Strongly Disagree

20. My appetite is as good as it was before the ostomy surgery. [Single-choice Question]

○ Strongly Agree

○ Agree

○ Uncertain

○ Disagree

○ Strongly Disagree

21. I have been able to accept the change in my appearance caused by the ostomy. [Single-choice Question]

○ Strongly Agree

○ Agree

○ Uncertain

○ Disagree

○ Strongly Disagree
